# A Computational Approach to the Functional Screening of Genomes

**DOI:** 10.1371/journal.pcbi.0030174

**Published:** 2007-09-28

**Authors:** Davide Chiarugi, Pierpaolo Degano, Roberto Marangoni

**Affiliations:** 1 Dipartimento di Matematica e Informatica, Università di Siena, Siena, Italy; 2 Dipartimento di Informatica, Università di Pisa, Pisa, Italy; University of California San Diego, United States of America

## Abstract

Comparative genomics usually involves managing the functional aspects of genomes, by simply comparing gene-by-gene functions. Following this approach, Mushegian and Koonin proposed a hypothetical minimal genome, Minimal Gene Set (MGS), aiming for a possible oldest ancestor genome. They obtained MGS by comparing the genomes of two simple bacteria and eliminating duplicated or functionally identical genes. The authors raised the fundamental question of whether a hypothetical organism possessing MGS is able to live or not. We attacked this viability problem specifying in silico the metabolic pathways of the MGS-based prokaryote. We then performed a dynamic simulation of cellular metabolic activities in order to check whether the MGS-prokaryote reaches some equilibrium state and produces the necessary biomass. We assumed these two conditions to be necessary for a living organism. Our simulations clearly show that the MGS does not express an organism that is able to live. We then iteratively proceeded with functional replacements in order to obtain a genome composition that gives rise to equilibrium. We ruled out 76 of the original 254 genes in the MGS, because they resulted in duplication from a functional point of view. We also added seven genes not present in the MGS. These genes encode for enzymes involved in critical nodes of the metabolic network. These modifications led to a genome composed of 187 elements expressing a virtually living organism, Virtual Cell (ViCe), that exhibits homeostatic capabilities and produces biomass. Moreover, the steady-state distribution of the concentrations of virtual metabolites that resulted was similar to that experimentally measured in bacteria. We conclude then that ViCe is able to “live in silico.”

## Author Summary

The origins of life represent a fascinating problem that has been addressed using different approaches and a wide variety of technologies. A theoretical approach consists of inferring a possible oldest ancestor genome from a well-defined comparison of current ones. A crucial problem concerns the validation of the proposed genome. The direct solution of synthesizing such a genome in a laboratory is often extremely difficult, due to the great complexity of a biological cell. In this paper, we present an approach for evaluating the chances a hypothetical organism has to be really viable, relying on computer simulations. Our method is based on a certain formal language, through which we specify a whole metabolic network, and we study its dynamics, in particular for verifying if a living organism has some fundamental properties, e.g., homeostasis. This approach is not equivalent to a wet-lab one, but it allows for early pruning of most of the inconsistently designed hypothetical organisms, thus saving biologists time and resources.

## Introduction

The search for LUCA, the Last Unknown Common Ancestor, is an open problem in evolutionary theory, which has been addressed using many different approaches. After the completion of several bacterial genomes, some authors tried to infer a possible minimal genome ruling out of non essential genes from existing small bacterial genomes. Dispensable genes were detected using both wet-lab techniques (e.g., see [1,2]) and comparative genomics methods (e.g., see [3]). The implicit hypothesis underlying these approaches is that the ancestor genome is made of singular elements only, and therefore would have a minimum size. We are aware of the criticisms raised about this hypothesis (e.g., see [4,5]), but a discussion on this subject would be off-topic for the present paper. Instead, we shall examine how such a simplified organism can be inferred by a comparative genomics approach, specifically following Mushegian and Koonin [3]. They considered the two very small genomes of Haemophylus influenzae and *Mycoplasma genitalium,* and manually scanned the two correspondent gene lists, so as to remove any element that looked redundant for biological function. The final result of this work was the so-called Minimal Gene Set (MGS), made of 254 singular genes (the original paper declared 256 genes, but two genes, -mg297 and mg336-, have been counted twice).

This hypothetical minimal genome was claimed to specify for a very essential prokaryote, but no argument was provided to address the fundamental question of whether a cell equipped with MGS (call it MGS-prokaryote) is able to live or not.

A direct, biological approach to answer this question could consist in synthesizing this genome, in cloning it in a ghost bacterium, and in evaluating the overall cell viability. However, there are many severe technical problems along this way, which make it hard to get an answer quickly.

We instead described this hypothetical cell as a computer program and simulated its behavior in silico. We then tested whether it shows some fundamental properties of living organisms. First of all we checked whether it enjoys homeostasis, i.e., the capability to reach a steady state in which the concentration of all the chemical species inside the cell fluctuates within a narrow range. We also investigated the capability of a MGS-prokaryote to produce biomass.

To model the MGS-prokaryote, we used (a variant of) the *π-*calculus, a process calculus designed to specify concurrent processes, which has already been used to describe biological phenomena [6,7]. We represented a complete set of metabolites, metabolic pathways, etc., involving the genes of the MGS-prokaryote. We ran in silico simulations and we observed the concentration of fundamental metabolites (ATP, NADH, etc.), checking the trend of their time courses toward constant values.

## Methods

### The Enhanced π-Calculus

To specify the metabolites and their relationships in terms of biochemical reactions, we used an enhanced version of the π-calculus, which has already been shown to be suitable for describing biological entities [8,9]. We refer the reader to [10] and [11] for a complete presentation of the (enhanced) π-calculus, and here we survey very briefly its fundamentals.

The π-calculus was designed to express, run, and reason about *concurrent systems*. These are abstract systems composed of *processes*, i.e., autonomous, independent processing units that run in parallel and eventually *communicate*, by exchanging *messages* through *channels*. A biochemical reaction between two metabolites, catalyzed by an enzyme, can be modeled in *π-*calculus as a communication. The two metabolites are represented by two processes, and, in our approach, the enzyme is modeled as the channel which permits the communication.

In addition to communications, the *π-*calculus also allows us to specify silent internal actions, used to model those activities of the cell, the details of which we are not interested in (e.g., the pure presence of a catalyst in a reaction, where it is not actively involved). The calculus has the means to express alternative behavior, when a metabolite can act in different possible manners: the way to follow is chosen according to a given probability distribution.

The main difference between the standard π-calculus and the enhanced version we used in this work is the notion of *address*. An address is a unique identifier of a process, totally transparent to the user, automatically assigned to all of its child subprocesses. This labeling technique helps in tracking the history of virtual metabolites and reasoning about computations, in a purely mechanical way. In particular, stochastic implementation or causality are kept implicit and are recovered as needed.

The enhanced π-calculus shares with other language-based approaches a number of advantages with respect to other formal descriptions. The very specification of the cell is actually a program and can be executed, giving rise to a virtual experiment, unlike other static descriptions such as the SBML [12]. Additionally, specifications turn out to be rather compact when compared, for example, with those expressed by P-systems [13], which, however, also describe membranes and their activities that we neglect here. Also, the specification of a whole organism is given by composing its constituents in a remarkably straightforward way. This is sometimes not the case with other approaches, e.g., those based on Petri Nets and used since [14], that have a nice graphical notation, but lack a linguistic framework. For a survey on process calculi for modeling biological entities, see also [15].

### The Interpreter

We specified in the π-calculus all the elements of the molecular machinery of the cell. Each element is specified in isolation, only defining its potential interactions with the environment. Then these pieces are put together in a compositional, holistic fashion. We wrote an interpreter for the enhanced π-calculus in Java, and we used it to run simulations. Simulations play the role of virtual experiments, performed according to given different initial conditions. The input file contains the definitions of all the metabolites inside the cell, the initial inner concentrations of the metabolites, and the rates of enzymatic activities, derived from the available real experimental data. The interpreter stores and displays some information about the virtual experiment, typically the concentrations of all the virtual metabolites (i.e., the number of the corresponding processes) or the usage of the different enzymes (i.e., the number of accesses of each channel) at given instants. With the first output, we determined the time course of the concentration of any virtual metabolite during the simulation; with the second one, we inspected the usage rate of the enzymes specified in the definitions, and, therefore, we tested the presence of unused metabolic pathways.

## Results/Discussion

### Simulation of the MGS-Prokaryote

The MGS-prokaryote has been exhaustively described in the enhanced π-calculus. We represented the 237 genes, their relative products, and the metabolic pathways expressed and regulated by the genes, as the corresponding processes and channels. In particular: the Glycolytic Pathway, the Pentose Phosphate Pathway, the pathways involved in nucleotide, aminoacids, coenzyme, lipids, and glycerol metabolism. Moreover, MGS genes encode for a set of membrane carriers for metabolite uptake, including the PTS carrier. We placed this virtual cell in an optimal virtual environment, in which all nutrients and water were available, and where no problems were present in eliminating waste substances.

### Global Performance of the MGS-Prokaryote

**Figure 1:**
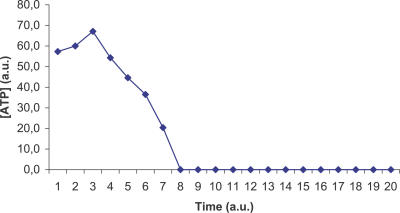
ATP Time Course for MGS Time course of the concentration of ATP in the MGS-prokaryote: this metabolite falls to zero in a short period of time. Abscissa: the simulated time; ordinate: the ATP concentration in arbitrary units.

**Figure 2:**
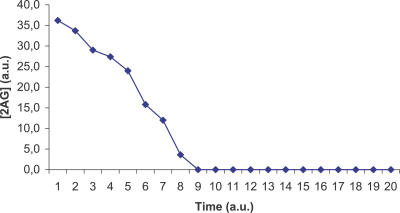
2AG Time Course for MGS Time course of the concentration of 2AG in the MGS-prokaryote: this metabolite falls to zero in a short period of time. Abscissa: simulated time; ordinate: 2AG concentration in arbitrary units.

A large number of simulations (about 5,000) have been run, differing in the values of the initial parameters. We independently varied the amount of glucose in the extracellular environment (the number of copies was in the range 100–5,000) and the time interval of observation T (in the range 10–10,000). Recall that in our model time steps correspond to the occurrence of transitions, so we set T establishing the length of the computations performed by the simulator. In all the studied cases, the MGS-prokaryote could not reach a steady state; most of the essential metabolites fell to zero in a short period, as is clearly shown in Figures 1 and 2, which display the typical time course of ATP and 2-Acyl-Glycerol (2AG).

These results lead us to the conclusion that this MGS-based cell was not able to live, at least in silico.

### Toward a Functionally Coherent Virtual Cell

#### An iterative approach to a minimal genome determination.

We approached the problem of establishing which genes present in the MGS were really necessary and which were missing for the cell's life. We manually inspected all the metabolic pathways, examining all the possible situations of missing or duplicated functions. In the case of a suspect functional deletion or duplication, we modified the MGS in two possible ways. On the one hand, we tried to recover a “broken pipe” by inserting the orthologous gene playing the requested function. The added gene is taken from the genome of *M. genitalium.* However, this is not always possible as M. genitalium is a parasite and is not expected to possess all the genes required for a free life. To find the missing gene, we then inspected the genome of H. influenzae and took the required gene from it. On the other hand, in the presence of a functional duplication or redundancy, we deleted one of the duplicated elements from the MGS, chosen on a case-by-case basis.

Once a modification had been made, we iteratively performed several simulations on the newly proposed genome, and we evaluated the time course of the metabolites.

Finally, our efforts converged to the genome of a hypothetical virtual cell, called ViCe. It is able to reach, after a while, a steady state in all its metabolites. Comparing the genome of ViCe with MGS, we note that the most important difference is due to the insertion in ViCe of seven genes which play fundamental roles (those beginning with “mg” come from *M. genitalium;* those beginning with “hi” come from H. influenzae): mg228, which encodes for Dihidropholate reductase, responsible for the reduction of the oxidized folate; hi0876, which encodes for Nucleoside diphosphate kinase, responsible for the phosphorylation of nucleosides diphosphate, using ATP as the phosphate donor; hi0874, which encodes for glycerol-3-phosphate acyltransferase, active in the early stage of complex lipids biosynthesis; mg069 and mg429, which encode for two components of the PTS system for glucose uptake; mg396, which encodes for ribulose-5-phosphate isomerase, a fundamental enzyme of Pentose phosphate pathway.

As said above, we ruled out 76 genes present in the MGS. We considered some of them dispensable, as their suppression seems to have no influence on the overall behavior of the virtual cell. Among the other excluded genes, there are mg049, mg382, and mg052 that encode for three enzymes involved in de novo nucleotide biosynthesis. In this case we felt free to rule them out because ViCe possesses the “salvage pathways” for nucleotide synthesis. The three enzymes mentioned above turned out to be functionally redundant and so did the correspondent genes.

#### The Virtual Cell.

The modifications described in the previous section resulted in the specification of the virtual prokaryote ViCe. It includes the following components:

1. A complete glycolytic pathway that allows the oxidation of glucose to pyruvate and reduced-NAD. Pyruvate is then converted to acetate which, being a catabolite, can diffuse out of the cell. A transmembrane reduced-NAD dehydrogenase complex catalyzes the oxidation of reduced-NAD; this reaction is coupled with the synthesis of ATP through the ATP synthase/ATPase transmembrane system. This set of reactions enables the cell to manage its energetic metabolism.

2. A Pentose Phosphate Pathway, composed of enzymes leading to the synthesis of ribose phosphate and 2-deoxyribose phosphate.

3. Enzymes for glycerol-fatty acids condensation, but no pathways for fatty acids synthesis. So, the latter metabolites must be taken from the outside.

4. The so-called “salvage pathways” for nucleotide biosynthesis. Thymine is the only nucleotide the cell is able to synthesize de novo.

5. A proper set of carriers for metabolites uptake: (a) a Glycerol Uptake Facilitator Protein; (b) a PtsG System for sugar uptake; (c) an ACP carrier protein for fatty acids uptake; (d) a broad specificity amino acids uptake ATPase; (e) broad specificity permeases for other essential metabolites uptake.

6. The necessary enzymes for protein synthesis, including DNA transcription and translation. The whole machinery necessary for DNA synthesis is also included in ViCe.

ViCe has no pathways for amino acid synthesis. All the necessary amino acids are uptaken from the external environment. All the nucleotide biosynthetic pathways are present in our model, so the cell is equipped with the basic means necessary for cell reproduction; however, at the present stage we have neither designed nor implemented in silico this activity. Some metabolites are considered to be ubiquitary, among which are water, inorganic phosphate, some metals ions, and Nicotinammide. Their concentration in an external or internal environment is assumed to be constant and not to be significantly affected by cellular metabolism.

Summing up, the cell can take metabolites from an external environment using the set of permeases and ATPases specified above. Among the pathways of our virtual cell, there is Glycolysis: glucose and fructose taken from the outside are oxidized yielding energy in the form of ATP and reduced-NAD. Pyruvate, the last metabolite of conventional Glycolysis, then becomes acetate, which, in turn, diffuses out of the cell. The cell “imports” fatty acids, glycerol, and some other metabolites, e.g., Choline, and uses them for the synthesis of triglycerides and phospholipids; these are essential components of the plasma membrane. Our virtual cell is also able to synthesize DNA, RNA, and proteins; the needed metabolites are mostly taken form the external environment or synthesized along its own pathways (e.g., Thymine and Ribose).

The detailed genome of ViCe, composed of 187 different genes, is listed in Table S1.

#### The performance of ViCe.

**Figure 3:**
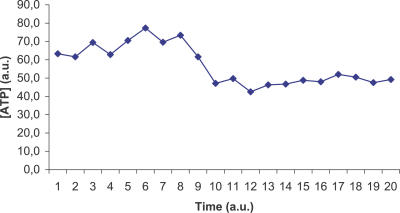
ATP Time Course for ViCe Time course of the concentration of ATP in ViCe: this metabolite reaches a steady state. Abscissa: simulated time; ordinate: ATP concentration in arbitrary units.

**Figure 4:**
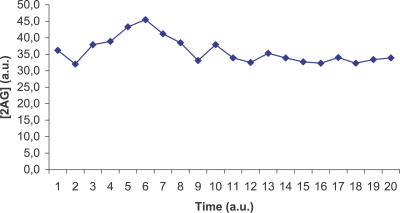
2AG Time Course for ViCe Time course of the concentration of 2AG in ViCe: this metabolite falls to zero in a short period of time. Abscissa: simulated time; ordinate: 2AG concentration in arbitrary units.

**Figure 5:**
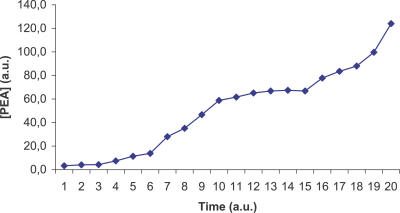
Phosphatidylethanolamine Time Course for ViCe Time course of the concentration of Phosphatidylethanolamine (PEA) in ViCe. The concentration of this metabolite increased with time. Abscissa: simulated time; ordinate: Phosphatidylethanolamine concentration in arbitrary units.

**Figure 6:**
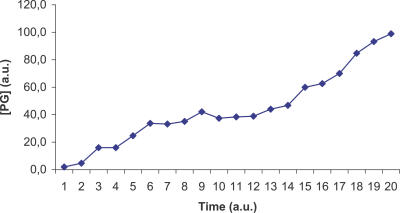
Phosphatidylglycerol Time Course for ViCe Time course of the concentration of Phosphatidylglycerol (PG) in ViCe. The concentration of this metabolite increased with time. Abscissa: simulated time; ordinate: Phosphatidylglycerol concentration in arbitrary units.

**Figure 7:**
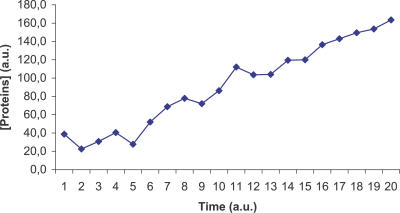
Protein Time Course for ViCe Time course of the concentration of the total amount of proteins in ViCe. The concentration increases with time. Abscissa: simulated time; ordinate: concentration in arbitrary units.

The ViCe genome is expressing a cell able to reach a steady state: Figures 3 and 4 display the time course of ATP and 2AG. To verify whether the trend of metabolites' time course reached the steady state (i.e., it approached a constant value), we performed a regression analysis. For each metabolite, we computed the average concentration value on the different experiments, at each time step. We then found that the distribution of this average versus time was definitively well approximated by a constant fit (linear with slope equal to 0), with r^2^ values ranging, among the different metabolites, from 0.79 to 0.91. The reader may wish to compare them with Figures 1 and 2, showing the same courses for the MGS-prokaryote. In addition, Figures 5–7 depict the time course of Phosphatydil ethanolamine, of Phosphatydil glycerol, and of the total protein quantity. We assumed the trend of these metabolites' concentration to be representative of the overall trend of the biomass. As shown in Figures 5–7, the concentrations of all the three kinds of metabolites grow with time. Then we can assess that ViCe's biomass increases, similarly to what happens to a real prokaryote.

**Figure 8:**
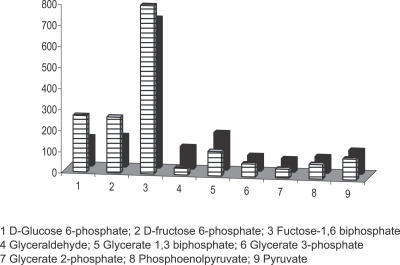
Relative Concentrations in ViCe and in the Real Case Relative concentrations of virtual (striped columns) and real (black columns) metabolites of the glycolysis pathway. The χ^2^ test reveals a significant (*p <* 0*.*05) overlap between the two distributions. The data relative to real concentrations are derived from [17].

Moreover, we considered the concentrations of the metabolites involved in the glycolytic pathway and we computed their respective proportions. We note that the results are compatible with those measured for real bacteria (see Figure 8 redrawn from [16], a companion paper focused on the computational aspects of ViCe). Therefore, ViCe is not only able to achieve homeostasis, but this equilibrium state turns out to be surprisingly “close” to that of a real cell.

#### A comparison with a novel wet-lab approach.

**Figure 9:**
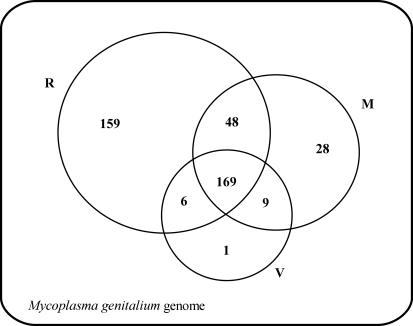
Intersections of the Reduced Genomes The genome of M. genitalium and the subsets of its genes considered to be not dispensable by: Glass et al., 6 (R-subset, composed of 382 genes); Mushegian and Koonin, 11 (M-subset, composed of 254 genes); and ViCe 1 (V-subset, composed of 185 genes), respectively. The remaining 50 genes are considered dispensable by all studies. We remark that the ViCe genome possesses two genes derived from H. influence which are not counted here.

Some authors recently used a wet-lab approach to characterize the minimum set of genes necessary to sustain bacterial life [1]. Their study involved *M. genitalium,* whose genome was supposed to be a close approximation of a minimal one. The experimentation consisted of knocking-out M. genitalium genes through global transposon mutagenesis. The resulting viable mutant strains were isolated and their genome analyzed to identify the disrupted genes. These genes were assumed to be dispensable for bacterial life. Below we compare the results of [1] with ours. The diagram depicted in Figure 9 shows the relationships between the set of genes considered not dispensable in [6] (call it R-genome), the MGS, and the genome of ViCe.

The intersection between the three sets contains 169 elements.

Because these genes resulted in not being dispensable according to all the three approaches, they are likely to be necessary for a minimal bacterium. Note that these genes represent 90% (169/187) of ViCe's genome. This can be seen as an argument for supporting the validity of our method. Moreover, consider that all but one of ViCe's genes (namely 186/187) are also included in R-genome or in MGS. In other words, the probability of obtaining a false positive (i.e., a necessary gene that resulted in not being dispensable according to the last two approaches) can be estimated to be on the order of 1/187.

Additionally, if we assume all the genes contained in the intersection of R-genome with MGS (namely 169 + 48) are not dispensable, the probability of obtaining a false negative, i.e., the probability of considering an essential gene as dispensable, can be estimated to be on the order of 48/187. Summing up, referring again to Figure 9, it turns out that the genes in ViCe number 187, and they are all essential. The genes present in both ViCe and in MGS and in R-Genome are 169; only one gene is in ViCe but not in R-genome or MGS, so the probability of a false positive is approximated by the ratio 1/187. The genes included in R-genome and in MGS but not in ViCe are 48, so the probability of a false negative is approximated by the ratio 48/187.

### Conclusions

Our approach to a functional screening of genomes was shown to be valid. In particular, our results have been obtained very cheaply with respect to a possible wet-lab approach involving de novo synthesis of the examined genome. Clearly, if a hypothetical genomes does not pass the in silico test, it will be unlikely to give rise to a living organism. It is hard to sustain the opposite: we cannot affirm that a hypothetical genome passing the test is able to sustain a living organism, and only a wet-lab approach can validate the proposal. Indeed, in silico experiments can help us to select which proposals are coherent, and thus more promising. As evidence of this, our work shows that the minimal genome we proposed for ViCe is surely more biologically reliable than an MGS.

## Supporting Information

Table S1 The ViCe's Genome Detailed list of the genome of ViCe.
(85 KB PDF)

## Abbreviations

2AG: 2-Acyl-Glycerol
MGS: Minimal Gene Set
ViCe: Virtual Cell
