# Supplementary material for: A Computational Approach to the Functional Screening of Genomes
Source: PLoS Comput Biol. 2007 Sep 28;3(9):e174. doi: 10.1371/journal.pcbi.0030174 (PMC1994977; doi:10.1371/journal.pcbi.0030174)
Supplement: Table S1 — Detailed list of the genome of ViCe. (85 KB PDF) [file pcbi.0030174.st001.pdf]

Glycolysis, pyruvate-to-acetate pathway, NADH oxidation, ATP synthesis

**Genes included in the *Minimal gene set***	**Encoded protein** *	**Genes included in ViCe**
mg050	§ fructose-1,6-bisphosphate (4.1.2.13)	mg050
mg058	§ phosphoglucoisomerase (5.3.1.9)	mg058
mg066	§ 6-phosphofructo-1-kinase	mg066
mg112	§ pyruvate kinase (2.7.1.40)	mg112
	§ pyruvate dehydrogenase complex	mg396
mg272	§ pyruvate dehydrogenase complex	mg272
mg273	§ pyruvate dehydrogenase complex	mg273
mg274	§ pyruvate dehydrogenase complex	mg274
mg300	§ phosphoglycerate kinase (2.7.2.3)	mg300
mg301	§ glyceraldehyde-3-phosphate dehydrogenase (1.2.1.12)	mg301
mg406	@ hypothetical protein	
mg407	§ enolase (4.2.1.11)	mg407
mg430	§ phosphoglycerate mutase	mg430
mg431	triose phosphate isomerase (5.3.1.1)	mg431
mg299	§ phosphate acetyl transferase (2.3.1.8)	mg299
mg357	§ acetate kinase (2.7.2.1)	mg357
mg275	§ NADH dehydrogenase [transmembrane complex]	mg275
mg398-405	§ ATP synthase complex [transmembrane complex]	mg398-405
mg053	phosphomannomutase (5.4.2.8)	mg053
mg063	° fructose-1-phosphate kinase (2.7.1.56)	mg063
mg119	@ Simple Sugar Transport System [ATP-binding protein]	mg119
mg120	@ Simple Sugar Transport System [permease protein]	mg120
mg187	§ multiple sugar transport system [ATP-binding protein]	mg187
	§ PTS System: PTSg [glucose-specific component]	mgo69
	§ PTS System: phosphotranferase (2.7.3.9)	mg429

Nucleotides metabolism

**Genes included in the *Minimal Gene Set***	**Encoded protein**	**Genes included in ViCe**
mg006	§ Timidilate kinase (2.7.4.9)	mg006
mg030	§ uracil phosphoribosyl tranferase	mg030
mg042-045	@ polyammine transport system (3.1.2.15)	mg042-045
mg049	§ purine nucloside phosphorilase (2.4.2.1)	
mg052	§ citidine deaminase (3.5.4.5)	
mg102	§ tioredoxine reductase (1.8.1.9)	mg102
mg107	§ guanilate kinase (2.7.4.8)	mg107
mg124	§ thioredoxin	mg124
mg125	@ hypothetical idrolase	mg125
mg127	° arsenate reductase (ArsC)	
mg171	§ adenilate kinase	mg171
mg227	§ timidilate synthase	mg227
mg229	§ ribonucleoside diphosphate reductase (1.17.4.1)	mg229
mg231	§ ribonucleoside diphosphate reductase (1.17.4.1)	mg231
mg268	° hypothetical protein	mg268
mg296	§ adenine phosphoribosyl transferase (2.4.2.7)	mg296
mg330	§ CMP kinase (2.7.4.14)	mg330
mg382	§ uridine kinase (2.7.1.48)	
mg434	putative uridilate kinase	mg434
mg438	° fructose-1-phosphate kinase (2.7.4.-)	mg438
	§ dihydropholate reductase	mg228
	nucleoside diphosphate kinase (2.7.4.6)	hi_0876

Aminoacids metabolism

**Genes included in the *Minimal Gene Set***	**Encoded protein**	**Genes included in ViCe**
mg077-080	aminoacid uptake complex	mg077-080
mg180	putative ATP-binding protein	
mg336	cysteine desulfurase 2.8.1.7	

mg391	putative leucine aminopeptidase (3.4.11.1)	

Lipids and glycerol metabolism

**Genes included in the *Minimal Gene Set***	**Encoded protein**	**Genes included in ViCe**
mg033	§ Glycerol uptake facilitator protein	mg033
mg038	§ glycerol kinase	mg038
mg114	§ PGP synthase	mg114
mg212	§ glycerol-3-phosphate acyl transferase (2.3.1.51)	mg212
mg287	§ Acil Carrier Protein	mg287
mg293	§ phosphodiersterase	mg293
mg333	@ ACP phosphodiersterase	mg333
mg437	CDP diglyceride sinthase (2.7.7.41)	mg437
	§ glycerol-3-phosphate acyl transferase (2.3.1.15)	hi0748

Coenzymes metabolism

**Genes included in the *Minimal Gene Set***	**Encoded protein**	**Genes included in ViCe**
mg013	§ methylenetetrahydrofolate dehydrogenase (1.5.1.5 3.5.4.9)	
mg047	§ S-adenosylmethionine synthetase 2.5.1.6	mg047
mg145	§ riboflavine kinase	mg145
mg245	° hypothetical protein	
mg270	§ lipoate peotein ligase	mg270
mg383	§ NAD+ synthase (6.5.3.1)	mg383
mg394	§ serine hydroxymethyltransferase (2.1.2.1)	mg394

Inorganic ions uptake

**Genes included in the *Minimal Gene Set***	**Encoded protein**	**Genes included in ViCe**
mg065	§ ABC:ATP carriers family	mg065

	dehydrogenase (1.5.1.5 3.5.4.9)	
mg071	@ hypothetical protein	mg071
mg322	@ hypothetical protein	mg322
mg410	§ Phosphate transfert system: permeasre pstA [ABC family]	mg410
mg411	§ Phosphate transfert system: permeasre pstA [ABC family]	mg411

Legenda:

§ Protein with functional role described in cyt
@ protein having a functional role considered uncertain or putative both in cyt and in KEGG.
° protein having a functional role considered uncertain or putative in cyt. In this case the annotation in cyt was corrected by us on the base of recent informations.
* numbers in parenthesys correspond to “Enzyme Commission numbers” of IUPAC-IUMB
